# Retrospective analysis of the diagnostic accuracy of lung ultrasound for pulmonary embolism in patients with and without pleuritic chest pain

**DOI:** 10.1186/s13089-022-00285-3

**Published:** 2022-08-12

**Authors:** Peiman Nazerian, Chiara Gigli, Angelika Reissig, Emanuele Pivetta, Simone Vanni, Thomas Fraccalini, Giordana Ferraris, Alessandra Ricciardolo, Stefano Grifoni, Giovanni Volpicelli, WINFOCUS and US SIMEU study group, Peiman Nazerian

**Affiliations:** 1 Careggi University Hospital, Florence, Italy; 2 San Giuseppe Hospital, Empoli, Italy; 3 SRH Poliklinik Gera GmbH, Pneumologische Praxis, Jena, Germany; 4 A.O.U. Città della Salute e della Scienza di Turin–Molinette University Hospital, Torino, Italy; 5 Emergency Medicine, San Luigi Gonzaga University Hospital, Regione Gonzole 10, Orbassano, 10024 Turin, Italy

**Keywords:** Pulmonary embolism, Lung ultrasound, Pleuritic pain, Diagnostic accuracy

## Abstract

**Background:**

Lung ultrasound (LUS) has a role in the diagnosis of pulmonary embolism (PE) mainly based on the visualization of pulmonary infarctions. However, examining the whole chest to detect small peripheral infarctions by LUS may be challenging. Pleuritic pain, a frequent presenting symptom in patients with PE, is usually localized in a restricted chest area identified by the patient itself. Our hypothesis is that sensitivity of LUS for PE in patients with pleuritic chest pain may be higher due to the possibility of focusing the examination in the painful area. We combined data from three prospective studies on LUS in patients suspected of PE and extracted data regarding patients with and without pleuritic pain at presentation to compare the performances of LUS.

**Results:**

Out of 872 patients suspected of PE, 217 (24.9%) presented with pleuritic pain and 279 patients (32%) were diagnosed with PE. Pooled sensitivity of LUS for PE in patients with and without pleuritic chest pain was 81.5% (95% CI 70–90.1%) and 49.5% (95% CI 42.7–56.4%) (*p* < 0.001), respectively. Specificity of LUS was similar in the two groups, respectively 95.4% (95% CI 90.7–98.1%) and 94.8% (95% CI 92.3–97.7%) (*p* = 0.86). In patients with pleuritic pain, a diagnostic strategy combining Wells score with LUS performed better both in terms of sensitivity (93%, 95% CI 80.9–98.5% vs 90.7%, 95% CI 77.9–97.4%) and negative predictive value (96.2%, 95% CI 89.6–98.7% vs 93.3%, 95% CI 84.4–97.3%). Efficiency of Wells score + LUS outperformed the conventional strategy based on Wells score + d-dimer (56.7%, 95% CI 48.5–65% vs 42.5%, 95% CI 34.3–51.2%, *p* = 0.02).

**Conclusions:**

In a population of patients suspected of PE, LUS showed better sensitivity for the diagnosis of PE when applied to the subgroup with pleuritic chest pain. In these patients, a diagnostic strategy based on Wells score and LUS performed better to exclude PE than the conventional strategy combining Wells score and d-dimer.

**Supplementary Information:**

The online version contains supplementary material available at 10.1186/s13089-022-00285-3.

## Background

Pleuritic chest pain, defined as a sharp chest pain exacerbated by breathing or coughing, is a common presenting symptom in the emergency department (ED) that requires a careful differential diagnosis between benign conditions such as musculoskeletal pain, and more serious diseases like pulmonary embolism (PE), pneumothorax, pneumonia with pleuritis and cancer [1]. Among these conditions, PE is a major cause of morbidity, mortality, and hospitalization [2]. In patients with PE, pleuritic pain is reported in up to 65% of patients in which distal emboli cause a pulmonary infarction [3]. However, diagnosing PE can be challenging in the ED because clinical signs and symptoms are non-specific. Moreover, definitive diagnosis often requires multidetector computed tomography pulmonary angiography (MCTPA) that is not feasible in unstable patients, it is not available 24 h a day in all institutions, it causes radiation exposure, and it can cause side effects due to contrast medium injection. For these reasons, while MCTPA remains the standard reference for PE, alternative bedside diagnostic tools that are non-invasive and readily available are of great importance in the clinical practice.

Point-of-care lung ultrasound (LUS) is a safe, rapid, and powerful bedside diagnostic tool with an acknowledged role in the diagnostic process of PE and several other conditions, particularly efficient when integrated in the clinical assessment of acute patients [4–7]. Some studies have shown that subpleural pulmonary infarcts can be detected by LUS as triangular, polygonal, or rounded pleural-based, echo-poor consolidations with sharp margins, with or without small localized pleural effusion [7, 8]. However, the role of LUS in the diagnosis of PE is limited to those cases in which MCTPA is contraindicated or not feasible [7]; indeed, its sensitivity for the diagnosis of PE is suboptimal when compared to MCTPA because subpleural infarcts are not always present in patients with PE [5, 7, 9–11]. Moreover, in patients with suspected PE, LUS may be challenging and laborious because the examination should be performed on the whole chest, as infarctions may be present in any chest area [8, 12].

On the other hand, a well localized pleuritic pain is often present in patients in which parietal pleura is involved in a peripheral pulmonary infarction. Therefore, focalizing LUS exam in the painful area indicated by the patient can simplify and make more effective the search for the infarcted areas of the lung [13–15]. To investigate these hypotheses, we analyzed existing data from three published studies to compare the diagnostic performance of LUS in two subgroups of patients with suspected acute PE, classified for presence or absence of pleuritic chest pain.

## Methods

### Study design and setting

We combined individual patient data from one prospective monocentric study, Reissig 2001 [16], and two prospective multicentric studies, Nazerian 2014 [12] and Nazerian 2017 [17], enrolling consecutive patients with suspected PE.

### Source study characteristics

After analysis of the existing literature on the topic, we selected for convenience the only 3 studies that reported complete information about the presence or absence of pleuritic pain on presentation. The studies had the following characteristics: (a) original publication; (b) prospective cohort study of patients with an objectively confirmed diagnosis of symptomatic PE; (c) record of presence or absence of pleuritic chest pain at presentation; (d) LUS performed in all enrolled patients.

### Source study quality assessment

One investigator, who was not a co-author of the three original studies included in the analysis, used the Quality Assessment of Studies of Diagnostic Accuracy included in Systematic Reviews-2 (QUADAS-2) tool to assess the methodological quality [18]. This tool is composed of two parts: risks of bias and concerns regarding applicability. The former was assessed in four domains patient selection, index test, reference standard and flow and timing, and the latter was assessed in three domains patient selection, index test and reference standard.

### Development of individual patient database

A core group of investigators (PN, GV and AR) developed the process for obtaining patient level data and the planned analyses, and all the co-authors approved them before the data collection phase.

After the investigators agreed to share their data, the databases were anonymously transferred to a central location under the auspices of PN. Data were checked, explanations for coding and uncertain data were clarified and a single pooled database was developed.

### Patient population

Reissig 2001 and Nazerian 2017 enrolled patients suspected of PE without differentiating the risk score, whereas Nazerian 2014 enrolled patients with Wells score > 4 (likely) or a positive d-dimer that underwent MCTPA.

The Wells score included the following items: clinical signs and symptoms of deep vein thrombosis (DVT) (+ 3), PE is most likely diagnosis or equally likely (+ 3), heart rate > 100 bpm (+ 1.5), immobilization at least 3 days or surgery in the previous 4 weeks (+ 1.5), previous objectively diagnosed PE or DVT (+ 1.5), hemoptysis (+ 1), malignancy with treatment within 6 months or palliative care (+ 1) [19]. Patients were categorized as PE likely if Wells score was > 4 and PE unlikely if ≤ 4. Cut-off values for d-dimer was < 500 ng/ml and not age-adjusted. All studies reported whether patients had pleuritic chest pain, that was defined as acute onset sharp pain exacerbated by breathing or coughing. Additional file 1: Table S1 reports LUS criteria for PE diagnosis and the reference test used in each study to formulate a final diagnosis of PE.

### Lung ultrasound

**Fig. 1:**
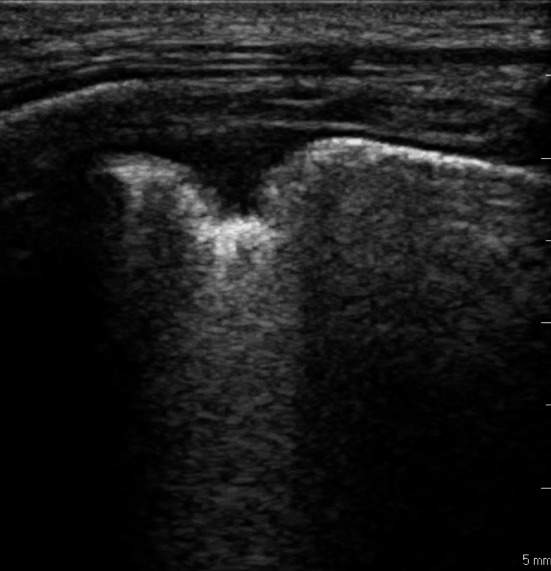
Image showing a typical pulmonary infarction as a wedge-shaped, pleural-based consolidation

In all studies LUS was performed by scanning the whole chest in 2 anterior, 2 lateral and 2 posterior chest areas per side; in each area, all the intercostal spaces were scanned searching for pulmonary infarctions. Details about the ultrasound machines and transducers used are reported in the method section of each study. Investigators performing LUS were blinded to diagnostic tests results and to all the clinical information except for symptoms of presentation and visible physical signs. The pattern considered positive for lung infarction was visualization of a pleural-based anechoic consolidation, wedge or round shaped, with sharp margins, without air bronchograms, of a minimum size measured at the pleural level of 0.5 cm with or without an associated small pleural effusion (Fig. 1). Two studies, Nazerian 2014 and Nazerian 2017, also reported the performance of a limited LUS examination based on a single ultrasonographic scan in the most painful chest area indicated by the patient.

### Statistical analysis

Data points are expressed as mean ± standard deviation. The diagnostic performance of LUS in all patients, and in patients with and without pleuritic chest pain was assessed by calculating accuracy (ROC curves), sensitivity, specificity, positive and negative predictive values, and likelihood ratios. The extended McNemar and the McNemar tests were used to compare sensitivities and specificities of LUS in patients with and without pleuritic chest pain, of global chest LUS examination approach versus a single LUS scan performed in the most painful area [20]. Using the same tests, we also compared two pre-test strategies for the prediction of PE: the combination between the clinical Wells score with the d-dimer test (Wells + d-dimer) versus the combination of the Wells score with the result of the LUS exam (Wells + LUS). The unpaired Student’s *t*-test was used to compare normally distributed data. Chi-square test was used for the comparison of variables expressed as proportions. To evaluate the most efficient strategy to rule-out PE, we compared the conventional approach recommended by international guidelines [2], i.e., Wells score unlikely (≤ 4) combined with negative d-dimer, to a LUS-based approach, i.e., Wells score unlikely combined with negative LUS. Efficiency is a statistical parameter well suited when diagnostic strategies based on a combination of different tests, are compared; it is the result of the number of true positive and negative test results of all positive and negative test results observed [21]. Failure rate (false negative proportion) of the Wells + d-dimer approach was calculated as the number of patients with a final diagnosis of PE in the group with Wells score ≤ 4 and negative d-dimer divided by all patients in the same group, whereas failure rate of the Wells + LUS approach was calculated as the number of patients with a final diagnosis of PE in the group with Wells score ≤ 4 and negative LUS divided by all patients in the same group. Efficiency of the Wells + d-dimer approach was calculated as the number of patients with Wells score ≤ 4 and negative d-dimer divided by all included patients, whereas efficiency of the Wells + LUS approach was calculated as the number of patients with Wells score ≤ 4 and negative LUS divided by all included patients. Efficiency of the Wells + LUS + dimer approach was calculated as the number of patients with Wells score ≤ 4, negative LUS and negative dimer divided by all included patients. Finally, we calculated the diagnostic accuracy of a third strategy: Wells + LUS + dimer (wells score unlikely, negative d-dimer, and negative LUS). A *p*-value < 0.05 indicates statistical significance. All *p*-values are two sided. Calculations were performed using SPSS and STATA statistical package (version 25.0, SPSS Inc., Chicago, Illinois, and version 13.0, STATA Corp, College Station, Texas).

## Results

### Source study with quality assessment, and patient characteristics

Among the 872 patients suspected of PE enrolled in the studies, 279 (32%) were diagnosed with PE. Additional file 1: Table S1 shows the derivation of the source studies used in this population-based analysis. The main characteristics of patients according to presence or absence of PE are shown in Table 1. Table 2 shows the leading diagnosis in all patients and in patients with and without pleuritic chest pain. D-dimer, measured in 808 patients, was positive in 340 out of 549 patients without PE (61.9%) and in 244 out of 259 with PE (94%, *p* < 0.001).

**Table 1:** Characteristics of the study population according to final diagnosis

	All patients (*n* = 872)	PE positive (*n* = 279)	PE negative (*n* = 593)	*P* value
Mean age ± SD	69.6 ± 16	69.2 ± 16	69.8 ± 15.8	0.549
Women	452 (51.8%)	151 (54.1%)	301 (50.8%)	0.354
Shock/hypotension	89 (10.2%)	23 (8.2%)	66 (11.1%)	0.189
Palpitations	85 (9.7%)	31 (11.1%)	54 (9.1%)	0.352
Dyspnea	561 (64.3%)	206 (73.8%)	355 (59.9%)	< *0.001*
Chest pain in general	310 (35.6%)	92 (33%)	218 (36.8%)	0.276
Pleuritic chest pain	217 (24.9%)	65 (23.3%)	152 (25.6%)	0.457
Signs and symptoms of DVT	205 (23.5%)	118 (42.3%)	87 (14.7%)	< *0.001*
Alternative diagnosis less likely than PE	427 (48.7%)	183 (65.6%)	244 (41.1%)	< *0.001*
HR > 100 bpm	302 (34.6%)	93 (33.3%)	209 (35.2%)	0.580
Immobilization or surgery	193 (22.1%)	59 (21.1%)	134 (22.6%)	0.630
Previous DVT or PE	122 (14%)	54 (19.4%)	68 (11.5%)	*0.002*
Hemoptysis	35 (4%)	11 (3.9%)	24 (4%)	0.942
Malignancy	169 (19.4%)	69 (24.7%)	100 (16.9%)	0.006
Wells score	3.5 ± 2.5	4.6 ± 2.6	2.9 ± 2.3	< *0.001*

Data are presented as number of cases (with %) or mean ±  SD =standard deviation

*DVT*   deep vein thrombosis, *PE  *pulmonary embolism, *HR*  heart rate

**Table 2:** Final diagnoses in all patients and in patients with and without pleuritic chest pain at presentation

	All patients *n* = 872	No pleuritic cp *n* = 655	Pleuritic cp *n* = 217
Pulmonary embolism	279 (32%)	214 (32.7%)	65 (30%)
Pneumonia	169 (19.4%)	121 (18.5%)	48 (22.1%)
Heart failure	80 (9.2%)	72 (11%)	8 (3.7%)
Musculo-skeletal chest pain	53 (6.1%)	11 (1.7%)	42 (19.4%)
COPD/pulmonary fibrosis	60 (6.9%)	55 (8.4%)	5 (2.3%)
Pleural effusion	38 (4.4%)	21 (3.2%)	17 (7.8%)
Syncope	37 (4.2%)	35 (5.3%)	2 (0.9%)
Tachyarrhythmia	32 (3.7%)	27 (4.1%)	5 (2.3%)
Acute coronary syndrome	19 (2.2%)	17 (2.6%)	2 (0.9%)
Lung cancer	20 (2.3%)	13 (2%)	7 (3.2%)
Psychogenic dyspnea	19 (2.2%)	16 (2.4%)	3 (1.4%)
Aortic dissection	6 (0.7%)	6 (0.9%)	0
Pericardial effusion	5 (0.6%)	3 (0.5%)	2 (0.9%)
Miscellaneous	55 (6.3%)	44 (6.7%)	11 (5.1%)

*COPD*  chronic obstructive pulmonary disease

Five patients with wells score ≤ 4 (unlikely) and negative d-dimer had a final diagnosis of PE; 4 out of these 5 patients presented with pleuritic chest pain. Additional file 1: Table S2 shows the quality assessment of each study.

### Lung ultrasound examination

Table 3 reports true positive, false positive, true negative and false negative results of LUS in the overall population and in patients with and without pleuritic pain. Table 4 reports the derived diagnostic performance of LUS in the same groups of patients. Sensitivity of LUS for the diagnosis of PE in patients with pleuritic chest pain (81.5%, 95% CI 70–90.1) was superior to patients without pleuritic chest pain (49.5%, 95% CI 38.8–77.6, *p* < 0.001), while specificity was similar (95.4% for patients with pleuritic chest pain and 94.8% for patients without, *p* = 0.86).

**Table 3:** Lung ultrasound in the diagnosis of pulmonary embolism

Population, *n*	True pos	False pos	True neg	False neg
All patients, 872	159	30	563	120
No pleuritic chest pain, 655	106	23	418	108
Pleuritic chest pain, 217	53	7	145	12
Scan in the most painful area, 156*	37	5	104	10

*pos* positive, *neg* negative

^*^Patients with pleuritic chest pain enrolled in the studies Reissig 2001 and Nazerian 2017

**Table 4:** Diagnostic performance of lung ultrasound for the diagnosis of pulmonary embolism in all patients and in patients without and with pleuritic chest pain

Population *n*	AUC	Sens	Spec	PPV	NPV	LR +	LR-
All patients 872	76 (72.1–79.8)	57% (51–62.9)	94.9% (92.9–96.6)	84.1% (78.1–89)	82.4% (79.4–85.2)	11.3 (7.83–16.20)	0.45 (0.40–0.52)
No pleuritic cp 655	72.2 (67.6–76.7)	49.5% (42.7–56.4)	94.8% (92.3–97.7)	82.2% (74.5–88.4)	79.5% (75.8–82.8)	9.50 (6.24–14.46)	0.53 (0.47–0.61)
Pleuritic cp 217	88.5 (82.6–94.4)	81.5% (70–90.1)	95.4% (90.7–98.1)	88.3% (77.4–95.2)	92.4% (87–96)	17.71 (8.51–36.84)	0.19 (0.11–0.31)
Scan in the painful area 156*	87.1 (79.7–94.4)	78.7% (64.3–89.3)	95.4% (89.6–98.5)	88.1% (75.6–99.6)	91.2% (85.7–94.7)	17.16 (7.2–40.92)	0.22 (0.13–0.39)

In brackets the 95% confidence interval

*AUC* area under the ROC curve, *Sens* sensitivity, *Spec* specificity, *PPV* positive predictive value, *NPV* negative predictive value, *LR* +   positive likelihood ratio, *LR*- negative likelihood ratio, *Pleuritic cp* pleuritic chest pain

^*^Patients with pleuritic chest pain enrolled in the studies by Reissig and Nazerian 2017

Considering the databases of the two studies reporting the findings of a simplified LUS exam focused on the painful chest area, this latter technique was applied in 156 patients. This simplified LUS showed similar sensitivity and specificity when compared to the whole chest LUS examination, respectively, 78.7%, 95% CI 64.3–89.3 vs 83%, 95% CI 69.2–92.3, *p* = 0.48 for sensitivity and 95.4%, 95% CI 89.6–98.5 vs 94.5%, 95% CI 88.4–98, *p* = 1 for specificity.

### *Strategies to rule-out PE: wells score* + *d-dimer vs wells score* + *LUS*

To evaluate the most efficient strategy to rule-out PE, we compared the conventional approach recommended by international guidelines [2], i.e., Wells score *unlikely* combined with *negative* d-dimer measurement, and a LUS-based approach, i.e., Wells score *unlikely* combined with *negative* LUS [17]. To this aim, data are derived from 451 patients enrolled in Reissig 2001 and Nazerian 2017 with available d-dimer, while Nazerian 2014 was not included because the study enrolled only patients with high pre-test probability of PE (Wells score *likely*) or a positive d-dimer. The overall analysis of these 451 patients, without differentiation for pleuritic pain, showed that the failure rate of the conventional approach was significantly lower when compared to the LUS-based approach (4.1% vs 12.4% *p* = 0.01) (Table 5). However, considering the subgroup of 141 patients complaining of pleuritic chest pain at presentation (Table 6), the LUS-based approach had a non-significant lower failure rate and higher sensitivity than the conventional approach (respectively, 3.7% vs 6.7%, *p* = 0.42 and 93% vs 90.7%, *p* = 1), but was significantly more efficient (56.7% vs 42.5%; *p* = 0.02) and more specific (78.6% vs 57.1%; *p* < 0.001).

**Table 5:** Comparison of different diagnostic strategies incorporating wells score, d-dimer measurement and LUS in 451 patients from Reissig 2001 and Nazerian 2017 studies

	-Wells score ≤ 4 -Negative d-dimer	-Wells score ≤ 4 -Negative LUS	-Wells score ≤ 4 -Negative d-dimer -Negative LUS
Failure rate*	4.1% (1.4–9.4)	12.4% (8.5–17.4)	0.9% (0.02–4.9)
Efficiency**	26.8% (22.8–31)	51.7% (46.9–56.4)	24.8% (20.9–29.1)
Sensitivity	96.6% (92.3–98.9)	80.5% (73.3–86.6)	99.3% (96.3–100)
Specificity	38.4% (32.9–44.1)	67.5% (61.9–72.8)	36.8% (31.3–42.5)
PPV	43.5% (41.2–45.8)	55% (50.6–59.5)	43.7% (41.5–45.8)
NPV	95.9% (90.6–98.2)	87.6% (83.9–91.2)	99.1% (94–99.9)
LR +	1.57 (1.43–1.72)	2.48 (20.07–2.97)	1.57 (1.44–1.71)
LR-	0.09 (0.04–0.21)	0.29 (0.21–0.41)	0.02 (0–0.13)

In brackets the 95% confidence interval

*LUS* lung ultrasound, *PPV* positive predictive value, *NPV* negative predictive value, *LR* +  positive likelihood ratio, *LR*- negative likelihood ratio

^*^Calculated as the number of patients within the group with a final diagnosis of pulmonary embolism divided by all patients in the same group

^**^Calculated as the number of patients within the group divided by all included patients

**Table 6:** Comparison of different diagnostic strategies incorporating Wells score, d-dimer measurement and LUS in 141 patients with pleuritic chest pain and available data on d-dimer, from Reissig 2001 and Nazerian 2017 studies

	- Wells score ≤ 4 - Negative d-dimer	- Wells score ≤ 4 - Negative LUS	- Wells score ≤ 4 - Negative d-dimer - Negative LUS	- Wells score ≤ 4 - Negative LUS (painful area)
Failure rate*	6.7% (1.9–16.2)	3.7% (0.8–10.6)	0% (0–6.5)	4.9% (1.36–12.2)
Efficiency**	42.5% (34.3–51.2)	56.7% (48.1 -65)	39% (30.9–47.6)	57.4% (48.8–65.7)
Sensitivity	90.7% (77.9–97.4)	93% (80.9–98.5)	100% (91.8–100)	90.7% (77.9–97.4)
Specificity	57.1% (46.7–67.1)	78.6% (69.1–86.2)	56.1% (45.7–66-1)	78.6% (69.1–86.2)
PPV	48.1% (42–54.3)	65.6% (55.4–73.7)	50% (44.4–55.6)	65% (55.7–73.3)
NPV	93.3% (84.4–97.3)	96.2% (89.6–98.7)	100%	95.1% (88.3–98)
LR +	2.12 (1.65–2.71)	4.34 (2.95–6.4)	2.28 (1.82–2.85)	4.23 (2.86–6.26)
LR-	0.16 (0.06–0.42)	0.09 (0.03–0.27)	0	0.12 (0.05–0.3)

In brackets the 95% confidence interval

*LUS* lung ultrasound, *PPV* positive predictive value, *NPV* negative predictive value, *LR* +  positive likelihood ratio, *LR*- negative likelihood ratio

^*^Calculated as the number of patients within the group with a final diagnosis of pulmonary embolism divided by all patients in the same group

^**^Calculated as the number of patients within the group divided by all included patients

Considering a strategy combining Wells score with a simplified LUS examination on a single focused scan in the most painful area, the above-listed results were confirmed, with lower failure rate and higher sensitivity that did not reach statistical significance (respectively, 4.9% and 90.7%, *p* = 0.65and *p* = 1), and significantly higher efficiency and specificity (respectively, 57.4% and 78.6%, *p* = 0.01and *p* < 0.001) compared to Wells score + d-dimer.

## Discussion

Our retrospective analysis shows that when patients suspected of PE complain of pleuritic chest pain, LUS searching for pulmonary infarction is a highly sensitive diagnostic tool that can provide useful information for ruling out PE.

Pleuritic pain is a frequent complaint in patients presenting to the ED; therefore, it is of the utmost importance distinguishing directly at the bedside between benign and potentially life-threatening causes of pleuritic pain. The first step in the evaluation of these patients should be discriminating the main cause of the symptom, whether is due to a chest wall process or to a lung disease involving the parietal pleura [14, 15]. When a chest wall condition, usually of muscular or joint origin, is identified and the patient has no respiratory signs and symptoms, it is safe to abandon any concern for emergency. On the other hand, when a LUS examination suggests a pulmonary condition, further work-up is needed and should be oriented by the results of LUS.

The parietal pleura is innervated with somatic pain receptors supplied by the phrenic nerve. When the parietal pleura is involved by the acute lesion and the pain receptors are stimulated, the signals are rapidly transmitted, leading to sharp and localized pain. In contrast, the visceral pleura has an autonomic nerve supply that develops from internal organs; pain sensations, if any, are transmitted slowly and are characterized as dull, achy, and slightly localized. Thus, if acute sharp pain is due to a pulmonary condition, the lung lesion must extend up to the parietal pleura; this latter is the typical process cascade of peripheral lung infarctions. Subpleural pulmonary infarctions are secondary to the occlusion of a pulmonary artery by a clot that leads to a rapid breakdown of the surfactant system, which promotes atelectasis and transudation of fluid into the affected lung tissue. The consequence of this cascade is local loss of alveolar air that allows ultrasound waves to penetrate the affected lung parenchyma and visualize the consolidation process.

Previous studies have shown a high prevalence of subpleural pulmonary infarcts in patients diagnosed with PE [5, 8, 16]. The possibility to visualize infarcts by using bedside LUS is limited by the necessity to explore the whole pulmonary parenchyma, which may become a challenging task in emergency situations and in obese or non-cooperating patients; in these situations, sensitivity of LUS may be affected and become suboptimal. However, when a patient presenting with pleuritic pain can indicate the most painful area, LUS exam can be more focused, and the physician can correlate the pattern observed with the symptom. If LUS shows the typical pattern of a lung infarction, i.e., a wedge-shaped, pleural-based consolidation without vascularization, it is possible to rule-out a chest wall origin of the pain and the probability of PE becomes very high. On the contrary, when LUS shows a regular sonographic pattern, the pain may be correlated to an extrapulmonary condition and PE becomes less likely.

To our knowledge, this is the first study investigating the performance of LUS for the diagnosis of PE in patients with pleuritic pain. Our data are derived from a large cohort of patients enrolled in three prospective studies, of which two (Nazerian 2014 and Reissig 2001) were included in two previously published meta-analyses [9, 11]. In our study, sensitivity of LUS in the general population was lower compared to previous studies and meta-analyses [8–11, 16]. This difference could be explained by the fact that most of our patients were enrolled in the ED, where a complete LUS examination can be limited by technical difficulties and time constraints. However, in the group of patients with pleuritic pain, LUS sensitivity was quite close to what was obtained in other studies, and this optimal result does not change when LUS examination is performed on the whole chest or limited to a single scan in the most painful chest area.

In patients with suspected PE, international guidelines recommend the use of a combination of d-dimer with standardized clinical scores, such as the Wells score, to optimize the diagnostic process [2]. Nevertheless, in our study, the conventional approach with Wells score + d-dimer demonstrated suboptimal accuracy in the group of patients with pleuritic pain, with a not negligible frequency of false negative results. On the other hand, a LUS-based approach (Wells score + LUS) showed a better performance in terms of efficiency and specificity. It is important to pinpoint that this result is obtained both when LUS is performed on the whole chest and when the examination is limited to a single scan focused in the most painful area; in emergency settings, where time restriction and practical difficulties in LUS evaluation are often a real challenge, a single scan examination is easier to perform and less time-consuming.

## Limitations

The main aim of our study does not coincide with the endpoint of the original studies retrospectively analyzed and included in our investigation. Moreover, we do not have complete data on the false negative studies to analyze better the kind of PE missed. Indeed, the original studies were designed to assess the diagnostic accuracy of LUS in the diagnosis of PE and did not specifically explore patients complaining of pleuritic pain. However, the populations studied were consecutively enrolled and well stratified for the presenting symptoms, including acute pleuritic chest pain.

We did not consider that patients presenting with severe respiratory failure and hemodynamic instability over pleuritic pain may benefit less from a bedside LUS searching for lung infarctions. In these cases, and in patients in whom another cause of chest pain cannot be excluded (like aortic dissection or other pulmonary pathologies), a multiorgan ultrasound evaluation and, if necessary, integration with other imaging tools like CT, represent safer strategies. Stratification of the risk should always guide the diagnostic approach in severe cases, but LUS for pleuritic pain in combination with a multiorgan evaluation may become strategic and improve predictivity [17]. New studies should be designed to respond to this question.

The three studies that we analyzed were multicentric and involved several operators; however, LUS was performed by well experienced physicians. Even if LUS is a simple technique with a steep learning curve, we cannot exclude that application of the same methodology by physicians with a lower skill level may result in different accuracy and safety. Finally, LUS examinations were conducted both with convex and linear probes. The linear probe allows better visualization of the subpleural regions when compared to other probes, whereas the convex probe is more panoramic. Using linear or convex probe separately may bring on variability. It is important to consider that the three studies were conducted by using a combination of the two probes, which seems to be the best strategy to optimize accuracy.

## Conclusions

Sensitivity of LUS for the diagnosis of PE is increased when the patients present pleuritic chest pain. In patients suspected of PE with pleuritic chest pain, a diagnostic strategy based on Wells score and LUS, whether performed on the whole chest or limited to a single scan in the painful chest area, is more efficient for ruling out PE compared to the conventional strategy based on Wells score and d-dimer.

## Supplementary Information

**Additional file1: Table S1.** Characteristics of source studies. **Table S2.** Risk of bias of individual studies.

## Data Availability

Not applicable.

## Abbreviations

ED: Emergency department
LUS: Lung ultrasound
MCTPA: Multidetector computed tomography pulmonary angiography
PE: Pulmonary embolism
