# Supplementary material for: Retrospective analysis of the diagnostic accuracy of lung ultrasound for pulmonary embolism in patients with and without pleuritic chest pain
Source: Ultrasound J. 2022 Aug 12;14:35. doi: 10.1186/s13089-022-00285-3 (PMC9374850; doi:10.1186/s13089-022-00285-3)
Supplement: Supplementary file 1 — Additional file1: Table S1. Characteristics of source studies. Table S2. Risk of bias of individual studies. [file 13089_2022_285_MOESM1_ESM.doc]

**Additional file 1**

Table S1. Characteristics of source studies

| ***Study*** | ***Design*** | ***Population, n*** | ***Lung US criteria for PE diagnosis*** | ***Reference test***  ***for PE diagnosis*** | ***Blinded adjudication of diagnosis*** | ***Follow-up*** |  |
| --- | --- | --- | --- | --- | --- | --- | --- |
| Reissig  2001 (14) | Monocenter, prospective cohort study | Patients suspected of PE, 69 | At least one pulmonary infarct defined as well-demarcated wedge-shaped or rounded lesions | MCTPA or combination of other diagnostic tests or autopsy | Yes | Yes | |
| Nazerian 2014 (13) | Multicenter, prospective cohort study | Patients presenting to ED, suspected of PE and with a Wells score >4 or a positive D-dimer, 357 | At least one pulmonary infarct defined as pleural based, well-demarcated echopoor triangular or rounded consolidations of  at least 0.5 cm in size | MCTPA performed in ED | Yes | No | |
| Nazerian 2017 (15) | Multicenter, prospective cohort study | Patients presenting to ED with suspected PE, 446 | At least one pulmonary infarct defined as pleural based, well-demarcated echopoor triangular or rounded consolidations of  at least 0.5 cm in size | Second level imaging diagnostic test or autopsy | Yes | Yes | |

US: ultrasonography; ED: emergency department; CT: computed tomography;

Table S2. Risk of bias of individual studies

|  | Risk of bias |  |  |  | Applicability concerns | |
| --- | --- | --- | --- | --- | --- | --- |
| Study | Patient selection | Index test | Reference standard | Flow and timing | Patient selection | Index test |
| Reissig 2001 | ☹ | ☹ | ? | ☹ | ☹ | ☺ |
| Nazerian 2014 | ☺ | ☺ | ☺ | ☺ | ☺ | ☺ |
| Nazerian 2017 | ☺ | ☺ | ☺ | ☺ | ☺ | ☺ |

☺ low risk; ☹ high risk; ? unclear risk
